# The Role of C21orf91 in Herpes Simplex Virus Keratitis

**DOI:** 10.3390/medicina55120753

**Published:** 2019-11-20

**Authors:** Vilija Danileviciene, Reda Zemaitiene, Vilte Marija Gintauskiene, Irena Nedzelskiene, Dalia Zaliuniene

**Affiliations:** 1 Department of Ophthalmology, Medical Academy, Lithuanian University of Health Sciences, LT-44307 Kaunas, Lithuania; reda.zemaitiene@kaunoklinikos.lt (R.Z.); daliazal@yahoo.com (D.Z.); 2 Department of Immunology and Allergology, Medical Academy, Lithuanian University of Health Sciences, LT-44307 Kaunas, Lithuania; gintauskiene@yahoo.com; 3 Department of Dental and Oral Pathology, Medical Academy, Lithuanian University of Health Sciences, LT-44307 Kaunas, Lithuania; Irena.Nedzelskiene@lsmuni.lt

**Keywords:** herpetic keratitis, C21orf91, confocal microscopy

## Abstract

*Background and Objectives:* This paper aims to describe the single nucleotide polymorphisms (SNPs) of C21orf91 rs1062202 and rs10446073 in patients with herpetic keratitis by evaluating corneal sub-basal nerves, as well as the density of Langerhans cells (LC) and endothelium cells (EC) during the acute phase of the disease. *Materials and Methods*: A prospective clinical study included 260 subjects: 70 with herpetic eye disease, 101 with previous history of herpes labialis—but no history of herpetic eye disease—and 89 with no history of any herpes simplex virus (HSV) diseases. All subjects underwent a complete ophthalmological examination including in vivo laser scanning confocal microscopy (LSCM) of the central cornea. C21orf91 rs1062202 and rs10446073 were genotyped using the real-time polymerase chain reaction (PCR) method with the Rotor-Gene Q real-time PCR quantification system. SNPs were determined using TaqMan genotyping assay, according to the manufacturer’s manual. *Results*: The C21orf91 rs10446073 genotype GT was more frequent in the HSV keratitis group, compared with healthy controls (20.0% vs. 7.9%), OR 2.929[1.11–7.716] (*p* < 0.05). The rs10446073 genotype TT was more frequent in healthy controls (12.4% vs. 1.4%), OR 22.0[2.344–260.48] (*p* < 0.05). The rs10446073 genotype GT increased the risk of EC density being less than 2551.5 cell/mm^2^, OR 2.852[1.248–6.515] (*p* < 0.05). None of the SNPs and their genotypes influenced the LC density and corneal sub-basal nerve parameters (*p* > 0.05). *Conclusions*: Our study reports a new association between herpetic keratitis and human gene C21orf91, with the rs10446073 genotype GT being more common in herpetic keratitis patients and increasing the risk for the disease by a factor of 2.9.

## 1. Introduction

HSV is widespread in the human population. Anti-HSV antibodies are found in about 88% of the human population at the age of 40 years [1]. Herpetic eye disease affects about 1% and herpes labialis about 40% of HSV-1 infected humans. After the first episode, the herpetic eye disease recurs during the first year for 10% of patients, within five years for 36% and within 20 years for 60% of patients [2]. Herpetic keratitis is a major cause of corneal blindness in developed countries [3].

Herpes labialis manifests itself in about 40% of HSV-1 infected people. For one-third of these, clinical manifestation recurs and for 50%, it recurs at least twice a year [2].

The C21orf91 gene, also known as the Cold Sore Susceptibility Gene 1, is located on chromosome 21 and is associated with herpes labialis manifestation [4,5]. The region includes a position from 15.7 to 18.6 Mb. The C21orf91 gene encodes a cytosolic protein, which is called the early undifferentiated retina and lens protein, but its function is still not clear. It is unknown whether the effect is concerned with the production of more or less of the encoded protein. The ways in which the C21orf91 gene influences pathogenesis and manifestation of herpes labialis have not been established yet [5]. The C21orf91 gene plays a role in biological processes, such as cerebral cortex neuron differentiation, cell differentiation and regulation of dendritic spine development. It is expressed within the developing central nervous system of mice and humans [6]. Researchers have found a connection between the C21orf91 gene and herpes labialis between the gene and Down syndrome, as well as hepatocellular carcinoma [4,5,6,7].

This is the first study tasked with finding any possible associations between the Cold Sore Susceptibility Gene and herpetic eye disease. The aim of our study was to determine whether the C21orf91 gene has an effect on herpetic keratitis manifestation, frequency, corneal morphological changes, corneal sensitivity, tear secretion and intraocular pressure during the acute phase of the disease.

## 2. Materials and Methods

A prospective clinical study included 260 subjects (167 women (64.2%) and 93 men (35.8%), mean age 59.1 ± 12.0 years, range 28.1–84.8) and were divided into three groups: 70 patients (35 women (50.0%) and 35 men (50.0%), mean age 66.6 ± 15.4 years, range 28.1–76.0) with active unilateral herpetic eye disease, 101 controls (72 women (71.3%) and 29 men (28.7%), mean age 58.9 ± 8.5 years, range 43.0–84.8) with previous history of herpes labialis, but no history of herpetic eye disease; and 89 healthy controls (60 women (67.4%) and 29 men (32.6%), mean age 58.5 ± 7.9 years, range 44.0–80.3) with no history of any HSV diseases.

Patients with epithelial blisters filled with fluid and dendritic infiltrates had epithelial keratitis; patients with stromal opacities, ulcerative infiltrates and/or neovascularisation had stromal keratitis; and with the ring infiltrate, cornea swelling around and precipitate on corneal endothelium had endothelial keratitis. Participants with a history of a burning pain followed by small blisters or sores on the lip margin were included in herpes labialis group.

Patients with previous history of other ocular infections, trauma, wearing contact lenses, diabetes mellitus, glaucoma and previous intraocular or refractive surgery were excluded from the study.

The study was approved by the Biomedical Research Ethics Committee (2015-07-09 Nr. BE-2-26 and 2017-01-26 Nr. P1-BE-2-26/2015). Written informed consent was obtained from all subjects who participated in the study.

All participants underwent a complete ophthalmological examination of both eyes and LSCM (Heidelberg Retina Tomograph 3 with the Rostock Cornea Module, Heidelberg Engineering GmbH, Dossenheim, Germany) of the central cornea. Images of all the layers of the cornea were obtained and three most representative images of each corneal layer were selected for counts of Langerhans cells (LC), endothelium cells (EC) and sub-basal corneal nerves. LC and EC were counted manually within a region of standardized dimensions (250 × 250 μm) and the result was given as cells/mm^2^ (cell/mm^2^) [8]. The corneal nerve analysis was performed using automated Corneal Nerve Fibre Analyser A CCMetrics V.2. The software automatically quantified the corneal nerve fibre length by the total length of nerves mm/mm^2^; nerve fibre density by the number of fibres/mm^2^; nerve branch density by the number of branch points on the main fibres/mm^2^; and nerve fibre total branch density by the total number of branch points/mm^2^ [9,10,11,12,13,14,15].

The C21orf91 rs1062202 and rs10446073 were genotyped using real-time PCR method with Rotor-Gene Q real-time PCR quantification system. The single nucleotide polymorphisms (SNPs) were determined using TaqMan genotyping assay, according to the manufacturer’s manual. Reagents were stored at −20°C until used.

The statistical analysis was performed with IBM SPSS statistics (Version 23.0, Chicago, IL, USA) programme. All parametric data were expressed as the mean and standard deviation. The Kolmogorov–Smirnov test was used for determination of quantitative data distribution. The results were analysed by Kruskal–Wallis and Mann–Whitney tests. The Kruskal–Wallis test was applied to compare the scores of more than two independent groups and the Mann–Whitney test for the scores of two independent groups. Differences on dependent variables were analysed by the Wilcoxon signed-rank test. χ^2^ tests were used for comparing frequencies of qualitative variables. In order to assess minimally false negative and minimally false positive results with the greatest accuracy, the method of ROC (receiver operating characteristics) curve was used. Logistic regression analysis was performed to determine the odds ratio predictive value. Differences were considered statistically significant when *p* values < 0.05.

## 3. Results

Data on 70 patients with active herpetic eye disease, including epithelial (n = 41), stromal (n = 14) or endothelial keratitis (n = 15), 101 subjects with previous history of herpes labialis, and 89 healthy controls were analysed and compared. Demographic data of all groups and subgroups are presented in Table 1.

**Figure 1:**
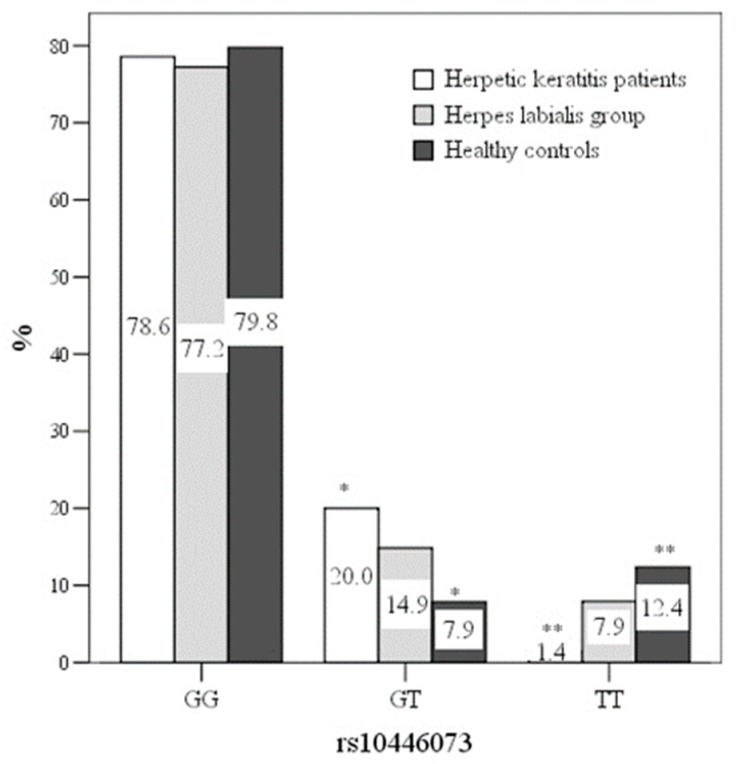
Prevalence of C21orf91 rs10446073 genotypes between groups. χ^2^ = 10.424, lls = 2, *p* = 0.034; * *p* = 0.03, ** *p* = 0.01.

The C21orf91 rs10446073 genotype GT was more frequent in the HSV keratitis group, compared with healthy controls (20.0% vs.7.9%), OR 2.929[1.11–7.716] (*p* < 0.05) (Figure 1). The rs10446073 genotype TT was more frequent in healthy controls (12.4% vs. 1.4%), OR 22.0[2.344–260.48] (*p* < 0.05). The C21orf91 rs1062202 genotypes were not statistically different between the groups (*p* > 0.05). None of the genotypes influenced the type of herpetic keratitis or the presentation of herpes labialis (*p* > 0.05). Data of the C21orf91 rs10446073 and rs1062202 genotypes are presented in Table 2.

EC density during the acute phase of HSV keratitis was higher in the rs10446073 genotype GG than in GT (2642.5+/-320.8 vs. 2487.8+/-464.5) (*p* < 0.05). The rs10446073 genotype GT increased the risk for EC density being less than 2551.5 cell/mm^2^, OR 2.852[1.248–6.515] (*p* < 0.05). LC and corneal sub-basal nerve parameters did not differ between the rs10446073 genotypes (*p* > 0.05). The rs1062202 genotypes did not influence any of corneal confocal microscopy parameters (*p* > 0.05) (Table 3).

The C21orf91 rs1062202 and rs10446073 did not influence clinical presentation of herpes labialis and its frequency (*p* > 0.05).

## 4. Discussion

JD Kriesel et al. have previously identified a region of chromosome 21 being significantly linked to herpes labialis disease. This region was defined using a genome-wide, family-based linkage study for human genes that may be linked to frequent herpes labialis [4]. They conducted SNP scans of the chromosome 21 region to define which of the six possible candidate genes were associated with cold sore frequency and identified the C21orf91 as a gene for susceptibility to herpes labialis [4]. JD Kriesel et al. demonstrated a connection between the C21orf91 gene genotypes and cold sore outbreaks, lifetime cold sores and cold sore severity in an unrelated human population [5]. They found some allele groups to be protective, some to provoke the disease with more frequency and more severe episodes. In this study, we described the effects of SNPs of C21orf91 rs1062202 and rs10446073 in patients with clinically active herpetic keratitis by evaluating corneal sub-basal nerves, and LD and EC density. The novelty of the study was to determine whether the C21orf91 gene had an effect on herpetic keratitis manifestation and corneal morphological changes during the acute phase of the disease.

In our study, we found that the C21orf91 rs10446073 genotype GT was more common in herpetic keratitis patients and increased the risk for the disease by a factor of 2.9. It also increased the risk by a factor of 2.9 for lesser EC density (<2551.5 cell/mm^2^) with more severe herpetic keratitis, especially in herpetic endotheliitis cases. Kriesel with colleagues found the rs10446073 genotype GT having an association with herpes labialis [4].

Based on our results, we did not find any correlations between the C21orf91 rs1062202 genotypes and herpetic keratitis, though JD Kriesel et al. found the rs1062202 genotype TC strongly associated with herpes labialis frequency [4].

JD Kriesel et al. in their family-related study combined alleles rs10446073 and rs1062202 to haplotype H5 and found that it was more common in frequently affected herpes labialis patients [4]. In our study, we did not find that the rs10446073 or rs1062202 genotypes would influence herpes labialis presentation, type of herpetic keratitis, LC and corneal sub-basal nerve parameters during clinically active herpetic keratitis.

For virus manifestation and reactivation, the virus access to the body and its latency are important, as are environmental factors and host genetics. Our study showed associations between the C21orf91 polymorphisms and manifestation of herpetic keratitis. This proved once again that the host genetics is important for virus manifestation and reactivation: the C21orf91 gene is important not only for herpes labialis, as shown in SD Kriesel study, but also for herpetic eye disease presentation.

To date, no relationship between HSV type 1 or type 2 and C21orf91 gene is known. JD Kriesel with colleagues has performed HSV-1 serotyping, but none of the authors have performed HSV PCR [4,5]. We were not able to perform PCR to detect HSV 1/2 and to establish associations between genotypes and HSV 1/2, which is a limitation of our study. We also evaluated only two alleles (rs10446073 and rs1062202) of the gene C21orf91 in a small, family-unrelated population, which could distort the results. More research is needed to assess the importance of C21orf91 gene polymorphisms for herpetic eye disease.

## 5. Conclusions

Our study reports a new association between herpetic keratitis and human gene C21orf91: rs10446073 genotype GT being more common in herpetic keratitis patients and increasing the risk for the disease.

## Figures and Tables

**Table 1:** Demographic data of patients with herpes simplex virus keratitis and control groups.

Parameters	HSV Keratitis			Total	Herpes Labialis Group	Healthy Controls
	Epithelial	Stromal	Endothelial			
Patients, n	41	14	15	70	101	89
Age (years) (mean ± SD)	62.2 ± 9.1	55.6 ± 13.2	56.4 ± 13.9	66.6 ± 15.4	58.9 ± 8.5	58.5 ± 7.9
Age range (years)	28.1–75.9	38.3–66.3	28.6–70.0	28.1–76.0	43–84.8	44–80.3
Gender, *n* (male/female)	28/13	3/11	4/11	35/35	72/29	60/29

There was no difference in age and gender between the groups (*p* > 0.05).

**Table 2:** Data of C21orf91 rs10446073 and rs1062202 genotypes prevalence between groups.

Parameters	HSV Keratitis			Total	Herpes Labialis Group	Healthy Controls
	Epithelial	Stromal	Endothelial			
Patients, n	41	14	15	70	101	89
rs1062202 AA	56.1	35.7	40.0	48.6	58.4	55.1
rs1062202 AG	36.6	64.3	46.7	44.3	35.6	37.1
rs1062202 GG	7.3	0.0	13.3	7.1	5.9	7.9
rs10446073 GG	75.6	58.7	80.0	78.6	77.2	79.8
rs10446073 GT	22.0	14.3	20.0	**20.0 ^a^**	14.9	**7.9 ^a^**
rs10446073 TT	2.4	0.0	0.0	**1.4 ^b^**	7.9	**12.4 ^b^**

Values reported as %. ^ab^ *p* < 0.05 (χ^2^ = 10.424, lls = 2).

**Table 3:** Quantitative analysis of LC, EC and corneal sub-basal nerve parameters in patients with different genotypes during acute phase of HSV keratitis.

Parameters	rs1062202			rs10446073		
	AA	AG	GG	GG	GT	TT
LC cell/mm^2^	141.7 ± 228.0	165.1 ± 221.6	148.46 ± 90.1	155.04 ± 229.3	155.64 ± 208.7	68.45 ± 54.9
EC cell/mm^2^	2648.74 ± 281.4	2606.4 ± 409.4	2545.4 ± 409.6	2642.53 ± 320.8 ^a^	2487.77 ± 464.5 ^a^	2698.37 ± 263.7
CNFD n/mm^2^	13.8 ± 12.6	12.8 ± 12.4	15.3 ± 10.3	13.2 ± 12.6	12.7 ± 11.4	17.8 ± 10.2
CNBD n/mm^2^	17.1 ± 21.7	15.6 ± 21.8	16.8 ± 13.8	16.2 ± 21.1	15.7 ± 20.2	20.9 ± 24.2
CNFL mm/mm^2^	10.9 ± 5.7	10.5 ± 5.7	11.2 ± 4.4	10.6 ± 5.8	10.9 ± 5.4	12.2 ± 4.4
CTBD n/mm^2^	34.9 ± 31.4	32.2 ± 31.7	31.9 ± 26.4	33.4 ± 30.8	32.3 ± 30.3	38.8 ± 36.8

Values reported as mean ± SD. *CNFD*—corneal nerve fibre density; *CNBD*—corneal nerve branch density; *CNFL*—corneal nerve fibre length; *CTBD*—corneal nerve total branch density. ^a^ *p* < 0.05, by Mann–Whitney test.
